# A Body Map Beyond Perceptual Experience

**DOI:** 10.5334/joc.347

**Published:** 2024-02-01

**Authors:** Daniele Gatti, Fritz Günther, Luca Rinaldi

**Affiliations:** 1 Department of Brain and Behavioral Sciences, University of Pavia, Pavia, Italy; 2 Institut für Psychologie, Humboldt-Universität zu Berlin, Berlin, Germany; 3 Cognitive Psychology Unit, IRCCS Mondino Foundation, Pavia, Italy

**Keywords:** mental representations, body representation, distributional semantics, semantic memory

## Abstract

The human body is perhaps the most ubiquitous and salient visual stimulus that we encounter in our daily lives. Given the prevalence of images of human bodies in natural scene statistics, it is no surprise that our mental representations of the body are thought to strongly originate from visual experience. Yet, little is still known about high-level cognitive representations of the body. Here, we retrieved a body map from natural language, taking this as a window into high-level cognitive processes. We first extracted a matrix of distances between body parts from natural language data and employed this matrix to extrapolate a body map. To test the effectiveness of this high-level body map, we then conducted a series of experiments in which participants were asked to classify the distance between pairs of body parts, presented either as words or images. We found that the high-level body map was systematically activated when participants were making these distance judgments. Crucially, the linguistic map explained participants’ performance over and above the visual body map, indicating that the former cannot be simply conceived as a by-product of perceptual experience. These findings, therefore, establish the existence of a behaviorally relevant, high-level representation of the human body.

## Introduction

The exact contribution of different experiential traces, such as perceptual and conceptual (i.e., linguistic) information, in organizing knowledge into mental representation is a topic of intense debate. Several authors in the past years, indeed, suggested that mental representations are essentially perceptual in nature and grounded on modal processing, but also that language (i.e., an abstract and amodal system) cannot approximate perceptual estimates (e.g., Barsalou, 2008). This view, generally labelled as Grounded (or Embodied) Cognition, gained popularity in the last decades as supported by several pieces of evidence (e.g., Calvo-Merino et al., 2005; Glenberg & Kaschak, 2002; Zwaan et al., 2002). However, evidence countering such a view at various levels is not lacking (Morey et al., 2022; but see also: Binder & Desai, 2011; Binder et al., 2009). Alternative perspectives, like the Symbol Interdependency Hypothesis (Louwerse, 2011; 2018; 2021), have in fact argued that cognitive processing relies on both perceptual and linguistic statistical information, with the access and use of a specific source of information depending on its availability and on contingent requests, set by the nature of the task at hand. For example, supporting this theory, it has been shown that language derived estimates (e.g., word frequencies) predict humans’ behavior when spatial stimuli are presented in non-iconic orientations (e.g., Boston – Seattle), but not in the reversed condition (Tillman et al., 2013), thus indicating that humans are able to flexibly rely on different set of (perceptual, conceptual, etc.) information depending on task requirements. Within this framework, the human body and, specifically, the way in which we mentally represent it, offers a unique opportunity to test these theoretical accounts.

Behavioral studies have reported a (spatial) distance effect when processing body part terms (e.g., Van Elk & Blanke, 2011), with pairs of words describing closer body parts (e.g., “nose – mouth”) being processed faster as compared with pairs of words describing body parts located farther apart (e.g., “ear – knee”). Evidence for a similar distance effect in body representation has been demonstrated when participants are asked to judge the relative distance between body parts (Smeets et al., 2009). Specifically, the distance effect would indicate that participants rely on an imagery strategy that in turn taps on an exploration of the human body reliant on visuospatial experience (for additional evidence reporting visuo-spatial and somatosensory involvement in body representation see: Noordzij & Postma, 2005; Peviani et al., 2019; Struiksma et al., 2011).

However, beyond the role of visuospatial experience, other experiential traces could participate as well in the exploration of the human body. In line with this possibility, recent neuroimaging evidence has shown that the representational structure of body maps in the lateral and ventral occipitotemporal cortices is mainly explained by functional and conceptual properties of body parts, rather than by visual and shape dimensions (Bracci et al., 2015). That is, the authors related the neural similarity matrix of the regions of interest (as emerging from a task in which participants viewed pictures of body parts) to the similarity matrices emerging from five different models indexing i) physical shape similarity, ii) perceived shape similarity, iii) physical proximity, iv) cortical homunculus similarity rankings, and v) semantic similarity of body parts as inferred from the frequency of body part word co-occurrence in large text corpora. Interestingly, the neural similarity matrices for pairs of body parts were best accounted by the semantic similarity between those same body parts, with this model outperforming all the other models (Bracci et al., 2015). Nevertheless, in the study by Bracci and colleagues’ (2015), the task was not tapping into the mental exploration of the body, but rather on body perception (i.e., passive view); as such, this leaves open the possibility that semantic processes could be less activated (or not activated at all) during an active spatial exploration of the human body. Furthermore, the measure indexing semantic processing used by Bracci and colleagues (2015) was reliant on the surface-level statistical structure of language (frequency of word co-occurrences, hence their contiguity) rather than on more comprehensive cues (the informativity of the relation between words, hence their contingency). Additionally, reanalyzing data collected by Jacobowitz (1973) and by Van Elk and Blanke (2011), it has also been demonstrated that body parts co-occurrence frequency predicts real body distance, with body parts co-occurring more frequently being rated as more similar and having lower physical distance (Tillman et al., 2012).

These latter findings point towards the possibility of co-existence of two experiential traces supporting body representation, a visuo-spatial one and a linguistic one. Building on this set of evidence, here we directly investigated whether these two experiential traces (i.e., perceptual and linguistic) could co-exist in human body representation by taking advantage of distributional semantic models (DSMs). DSMs induce words meanings from large databases of natural language data, representing them as high-dimensional numerical vectors: these models are indeed thought to well capture the structure of semantic memory (Günther, et al., 2019; Jones, et al., 2015). DSMs are trained on large corpora that document natural language use and estimate the meaning of a target word on the basis of the lexical contexts in which it appears (i.e., the words it co-occurs with in the text). The distributed representation, or vector, of a target word can be quantitatively compared with another by geometrically measuring the angle between the two vectors in a multidimensional space, which in turn is thought to capture semantic similarity between words (Günther et al., 2019): similar words will occur in similar contexts, ending up being associated with vectors that are geometrically closer. However, note that, despite this geometrical interpretation, the architecture of these models is entirely non-spatial (in the sense that it is not tied to the actual physical space in the outside world, but only to language information). Importantly, DSMs have been shown to be high-performing across a wide range of tasks tapping on semantic (e.g., Brown et al., 2023; Gatti, Marelli, & Rinaldi, 2022; Gatti, Rinaldi et al., 2022), and geographic spatial information (Gatti, Marelli et al., 2022). Moreover, they are equivalent to psychologically grounded associative learning models (Hollis et al., 2017; Mandera et al., 2017).

Specifically, in Experiment 1, we investigated to what extent linguistic distances extracted from DSMs resemble real spatial body distances across six different languages. Then, in Experiment 2, participants were shown the names of two body parts and were asked to indicate which one was closer to the eyes or the feet. Here, we investigated whether linguistic distances predict participants’ performance in this task over and above real distance between body parts. Finally, in Experiment 3, we adopted a non-linguistic task, in which participants were shown the images of two body parts and asked to indicate which was closer to the eyes or the feet.

In the two tasks used in Experiment 2 and Experiment 3, one could expect linguistic predictors to have little relevance in explaining participants’ performance. Namely, one could assume that, when asked to mentally explore their body in a non-action-oriented task (De Vignemont, 2010), participants would rely mainly on imagery processes (i.e., which in turn tap on visual experience), and thus one would expect only real body distance to play a central role. However, seminal studies have reported the existence of systematic distortions in the localization of body joints (Gurfinkel & Levick, 1991), thus raising the possibility that participants’ performance could be explained by a more complex model than the one comprising only spatial measures. Consistent with this, previous studies investigating geographic spatial representation have shown that language-based indexes (i.e., DSM-based) can outperform real spatial measures in predicting humans’ performance (Gatti, Marelli et al., 2022). These results point towards the possibility that language-based information could account for participants’ performance in a task tapping on spatial representations in general, including spatial body representation.

## Experiment 1

### Material and methods

#### Stimuli

**Figure 1:**
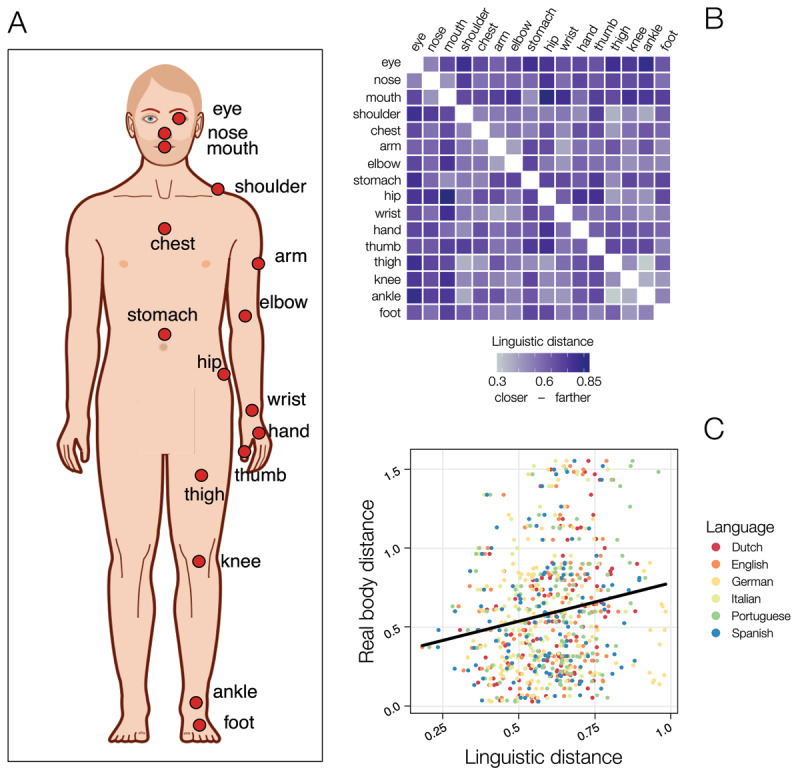
A graphical representation of the body parts included in Experiment 1 **(A)** note that this figure is only for graphical reasons, as it is not standard in its proportions). A heatmap representing the linguistic distances across body parts words in English **(B)**. The positive relationship between semantic and real body parts distances across the six languages tested **(C)**.

We selected 16 body part words (*eye, arm, stomach, chest, thumb, elbow, foot, hand, wrist, hip, knee, ankle, mouth, nose, thigh, shoulder*; Figure 1A) across six different languages (English, German, Dutch, Italian, Portuguese and Spanish). These words were translated from English in Dutch, German and Italian from native speakers, while for the other languages they were translated through NorthEuraLex (i.e., a vocabulary database; Dellert et al., 2019); in case of word unavailability in NorthEuraLex we used Google Translate. These body parts were selected in order to have a good distribution of items across the entire body. Critically, while selecting the items, words used in one language but not in others (e.g., the word “toe” cannot be translated in Italian with a single word) were excluded. We then computed the prototypical physical distance between these body parts as well as the linguistic distance as extracted from natural language.

From the standing human body figure (with arms parallel to the body trunk and legs aligned) reported by Panero and Zelnik (1979), using Adobe Photoshop CS (2004), we measured the distance among the selected body parts (i.e., each body parts pair, fzor a total of 120 pairs all lateralized from the same side when possible) and rescaled these values for a human body of 170 cm in height^1^ (please note that this was done in order to allow for better understanding of the effects, but the proportional relationships between the various items do not change when changing the overall height of the figure). The physical distances between body parts were labeled BDist (see Figure 1A for a list of the body parts included). To ensure that the body distances extracted from Panero and Zelnik (1979) were reliable, we also extracted the same metrics from a figure standing in anatomical posture (i.e., in which the thumbs point out from the body, see the two figures in the OSF at: https://osf.io/dnt2p/). The distances extracted from these two figures were extremely correlated, *r* = .99. As such, in our analyses we kept only the distances extracted from Panero and Zelnik (1979).

#### Distributional semantic model

Following a similar rationale as for physical distances, we computed the linguistic distances among the selected body parts for each of the six languages considered. The DSM used here was *fastText* (Bojanowski et al., 2017), and words vectors were retrieved from English, German, Dutch, Italian, Portuguese and Spanish pre-trained vectors (Grave et al., 2018). The models are trained on Common Crawl and Wikipedia using the Continuous Bag of Words (CBOW) method, an approach originally proposed by Mikolov and colleagues (2013), with 300 dimensions, character n-grams of length 5 and a window of size 5. When using CBOW, the obtained vector dimensions capture the extent to which a target word is reliably predicted by the linguistic contexts in which it appears.

All the DSMs included word vectors for 2 million words and a variable number of sub-word tokens. The English DSM included 500 billion tokens, the German DSM included more than 65 billion tokens, the Dutch DSM more than 17 billion tokens, the Italian DSM more than 36 billion tokens, the Portuguese DSM more than 35 billion tokens, and the Spanish DSM more than 72 billion tokens.

From these semantic spaces, we extracted vector representations for the words used in this study. Specifically, for each word pair we computed a linguistic-distance index (hence LDist) based on the cosine of the angle formed by vectors representing the meanings of these words (in terms of their distributional history in language) subtracted from 1: the lower the LDist value, the closer (i.e., more semantically similar) the words are in the semantic space as estimated by the model. For a graphical representation of linguistic distances between body parts in English see Figure 1B.

### Data analysis and results

All the analyses were performed using *R-Studio* (RStudio Team, 2015). We estimated a linear mixed model (LMM) using the *lme4* and *lmerTest* R packages (Bates et al., 2014; Kuznetsova et al., 2015) having body distances (hence BDist) as dependent variable, LDist as continuous predictor in interaction with the Language; items (with the same values across different languages; that is, for example, “hand” in English and its Italian translation “mano” were treated as the same item) were included as random intercept. In *lme4* syntax the tested model was:


\[
BDist\;\sim\;LDist*Language + \left( {1{\mathrm{|}}Item1} \right) + \left( {1{\mathrm{|}}Item2} \right)
\]

We then performed a model selection using the *MuMIn R* package, with the function *dredge* (Bartoń, 2020). This procedure selects the best fitting model (i.e., the one with lowest Akaike information criterion, which returns an estimation of the quality of the model, AIC; Akaike, 1973) fitting all the possible combinations of the fixed effects included (i.e., the model selection procedure was not allowed to drop the random effects).

The best model identified by the model selection procedure included only the effect of LDist, *b* = .45, *t*(704.43) = 5.11, *p* < .001, *Pseudo-R²* (total) = .55; *Pseudo-R²* (fixed effects) = .02 (Figure 1C). No other model had Δ AIC < 2 compared with the best model selected.^2^ The model selection thus dropped both the interaction term and the fixed effect of the Language. The best fitting model had AIC = 304.5 and the full model had AIC = 317.6 (Δ AIC = 13.05), thus indicating that the model including only LDist was 682 times more likely to be a better model as compared with the full one.

These results show that the linguistic distances of body parts as extracted from language resemble real body distances: more semantically distant body parts words are also physically more distant on the body surface. However, the portion of the variance explained is small enough to hypothesize that these two types of information (i.e., a spatial one and a semantic one) could be partially independent. To test this hypothesis, we thus performed a first behavioral experiment to probe whether both spatial and linguistic distance are simultaneously considered when humans have to classify the distance between pairs of body parts.

## Experiment 2

### Methods

#### Participants

Sample size was determined a-priori based on Brysbaert and Stevens’ (2018) indication that, in order to achieve properly power, an experiment should have at least 1,600 observations per cell of the design (i.e., per condition tested), that is at least 40 stimuli for 40 participants.

Forty Italian students (6 males, *M* participants’ age = 24, *SD* = 2.9) participated in the experiment. All participants were native Italian speakers, had normal or corrected to normal vision and were naïve to the purpose of the experiment. Informed consent was obtained from all participants before the experiment. The protocol was approved by the psychological ethical committee of University of Pavia and participants were treated in accordance with the Declaration of Helsinki.

#### Stimuli and procedure

From the 16 Italian words used in Experiment 1, we selected two body parts to be used as reference points (i.e., eyes and feet).^3^ Then, for each of the two reference points, we built all the possible word-pairs among the 15 remaining words (105 word-pairs; the word *foot* was kept in the *eyes* condition but not in the *feet* condition; analogously, the word *eye* that was kept in the *feet* condition but not in the *eyes* condition), which were also reversed for a total of 210 word-pairs (i.e., the two words were presented on the screen, one on the left and one on the right hemispace; we thus counterbalanced their spatial position on the screen). Participants performed the task twice in different days, one time with the *eyes* condition and another with the *feet* condition, in counterbalanced order across participants.

At the beginning of the experiment, participants were instructed to focus on a reference point, and then, in each trial they were shown a word-pair and asked to indicate which one of the two body parts was closer to the reference. Participants were instructed to respond as fast and accurately as possible by pressing the left/right key (A and L) using the index finger of the left and right hand, in order to indicate the body part placed on the left side or the one on the right side, respectively. The trials were shown in random order.

**Figure 2:**
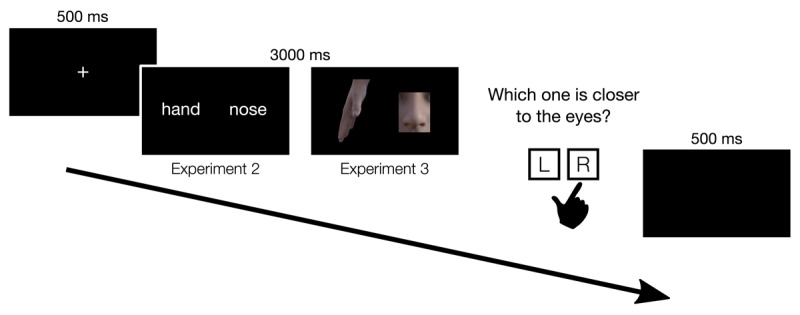
The timeline of events in Experiment 2 and Experiment 3. In Experiment 2, participants were shown with the names several pairs of body parts (e.g., hand – nose) and were asked to indicate which one was closer to the eyes or the feet (depending on the experimental condition). Experiment 3 was identical to Experiment 2, but in this case participants were shown images of body parts.

Each trial started with a central fixation cross (presented for 500 ms) followed by a word-pair (with the two words being completely in the two different halves of the screen) and then, after participants’ response or after 3000 ms, the trial moved to a black screen (presented for 500 ms) which ended the trial (Figure 2). Participants’ responses were recorded only during the 3000 ms of word-pair presentation.

At the end of the experimental task (in each condition) participants were asked to indicate in which position (i.e., standing, with different possible positions of the arms or sitting, see Supplementary Material) they imagined the body while performing the experiment.

Participants were tested online using Psychopy (Peirce, 2007, 2009; Peirce & MacAskill, 2018; Peirce et al., 2019) through the online platform Pavlovia (https://pavlovia.org/).

#### Distributional semantic model

The Italian DSM used was the same adopted in Experiment 1.

#### Computation of body and semantic predictors

For each word-pair we computed two predictors for quantifying physical and linguistic distances, respectively: ΔBDist and ΔLDist, both computed as the absolute value of the difference of BDist values or LDist values between the two body parts and the reference point, with small values indicating that the two body parts are placed at a similar distance to the reference point and higher distances indicating that there is one of the two substantially closer than the other. For both predictors, for each trial comprising the *k* and *j* body parts pair, the formula was:


\[
\Delta predictor = |Dist\left( {k,reference} \right) - Dist\left( {j,reference} \right)|
\]

#### Data analysis

All the analyses were performed using *R-Studio* (RStudio Team, 2015). LMMs and generalized LMMs (GLMMs) were run using the *lme4* R package (Bates et al., 2014; Kuznetsova et al., 2015). All the estimated models had random intercepts for participants and items. The plots reported were obtained using the *effects* R package (Fox, 2003; Fox & Weisberg, 2019). Our main dependent variable was participants’ correct log-transformed response times (RTs), which were analyzed using LMMs; we also analyzed participants’ accuracy, using GLMMs fitted on a binomial family distribution (i.e., correct answers were computed as 1s and wrong answers as 0s).^4^

For both RTs and accuracy, we performed the same analyses. Specifically, we estimated a model including additively ΔBDist and ΔLDist and their interaction with the reference (*eyes* vs. *feet*) as a within-participant factor. In *lme4* syntax, the model estimated was:


\[
DV\sim\left( {\Delta BDist + \Delta SDist} \right)*Reference + \left( {1{\mathrm{|}}Participant} \right) + \left( {1|left\;item} \right) + \left( {1|right\;item} \right)
\]

### Results

Trials in which overall RTs were faster than 300 ms or in which participants did not provide an answer within 3000 ms (2.5% of the trials) were excluded from the analysis. All the participants had accuracy >75%, and the mean error rate was = 14%.

**Figure 3:**
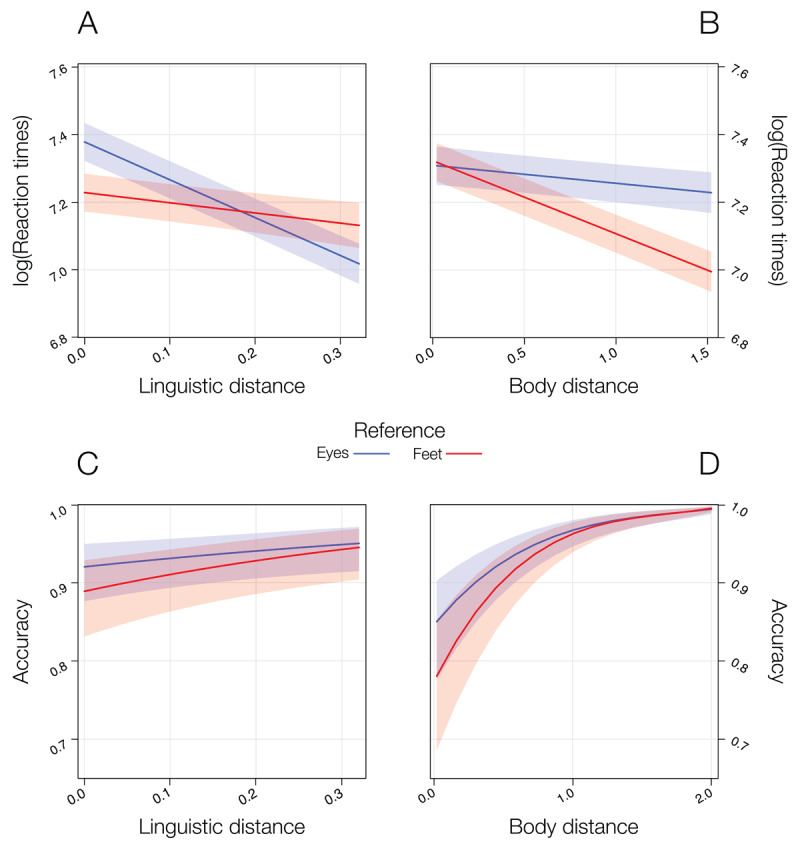
Plots of the significant effects observed in the statistical model comprising real body and linguistic distances in Experiment 2. For RTs, the higher the distances, the faster participants’ response, with this effect being moderated by the reference point. Specifically, the effect of linguistic distance is stronger in the *eyes* condition then in the *feet* condition **(A)**, while for body distance the opposite pattern was observed **(B)**. For accuracy, the higher the distance, the higher the participants’ accuracy for both semantic **(C)** and body distance **(D)** predictors.

The results of the LMM on RTs and of the GLMM on accuracy are reported in Table 1, and in Figure 3.

**Table 1:** Results of the LMM on RTs and of the GLMM on accuracy for Experiment 2.


FIXED EFFECT	*REACTION TIMES*			*ACCURACY*		
	
	*F*-VALUE	*NumDF, DenDF*	*p*-VALUE	χ^2^-VALUE	*DF*	*p*-VALUE

ΔBDist	213.5	**1,4092**	**<.001**	102.93	**1**	**<.001**

ΔLDist	240.6	**1,6216**	**<.001**	5.11	**1**	**.02**

Reference	28.1	**1,11955**	**<.001**	25.72	**1**	**<.001**

ΔBDist : Reference	111.5	**1,11288**	**<.001**	.72	1	.39

ΔLDist : Reference	74.2	**1,5527**	**<.001**	2.67	1	.10


Both predictors negatively predicted RTs (i.e., the higher the distances, the faster the RTs). Specifically, the significant interaction ΔBDist by Reference indicated that the negative effect of ΔBDist is stronger in the *feet* condition compared with the *eye* condition, but both are significant, *b* = –.22, *t*(8881) = –19, *p* < .001, and *b* = –.05, *t*(4699) = –4.21, *p* < .001, respectively. Conversely, the significant interaction ΔLDist by Reference indicated that the negative effect of ΔLDist is stronger in the *eyes* condition compared with the *feet* condition, but both are significant, *b* = –1.12, *t*(6613) = –21.33, *p* < .001, and *b* = –.30, *t*(5526) = –3.90, *p* < .001, respectively.

On accuracy, no interaction was found. The significant effects of ΔBDist and ΔLDist indicates that for both predictors, the higher the distance, the higher participants’ accuracy, *b* = 1.67, *z* = 10.15, *p* < .001, and *b* = 1.57, *z* = 2.26, *p* = .02, respectively. The significant effect of Reference indicates that participants’ accuracy was higher in the *eyes* condition compared with the *feet* condition, *b* = .54, *z* = 5.07, *p* < .001.

These results suggest that, while solving a task requiring participants to process distance-like information on the body surface, participants relied simultaneously on both visual and semantic knowledge. This in turn indicates that the linguistic body map cannot be considered simply as a by-product of the real body map. However, the semantic effect observed in the present experiment could depend on the fact that the task used here taps extensively on linguistic processes (i.e., the stimuli used were words). In order to provide more direct evidence for a semantic involvement in body representation we then performed a third experiment, this time employing body parts images instead of words.

## Experiment 3

### Methods

#### Participants

Forty-six Italian students participated in the study. Six participants were removed from the analyses after reporting that they imagined the body as sitting.^5^ The final sample included 40 participants (9 males, *M* participants’ age = 23, SD = 2.5). All participants were native Italian speakers, had normal or corrected to normal vision and were naïve to the purpose of the study. None of the participants had participated in Experiment 2. Informed consent was obtained from all participants before the experiment. The protocol was approved by the psychological ethical committee of University of Pavia and participants were treated in accordance with the Declaration of Helsinki.

#### Stimuli and procedure

From the 16 body parts used in Experiment 1, we dropped those that could not be represented clearly using an image; hence, the final set of body parts included 10 stimuli (*arm, chest, eye, foot, hand, knee, mouth, shoulder, stomach* and *nose*).

**Figure 4:**
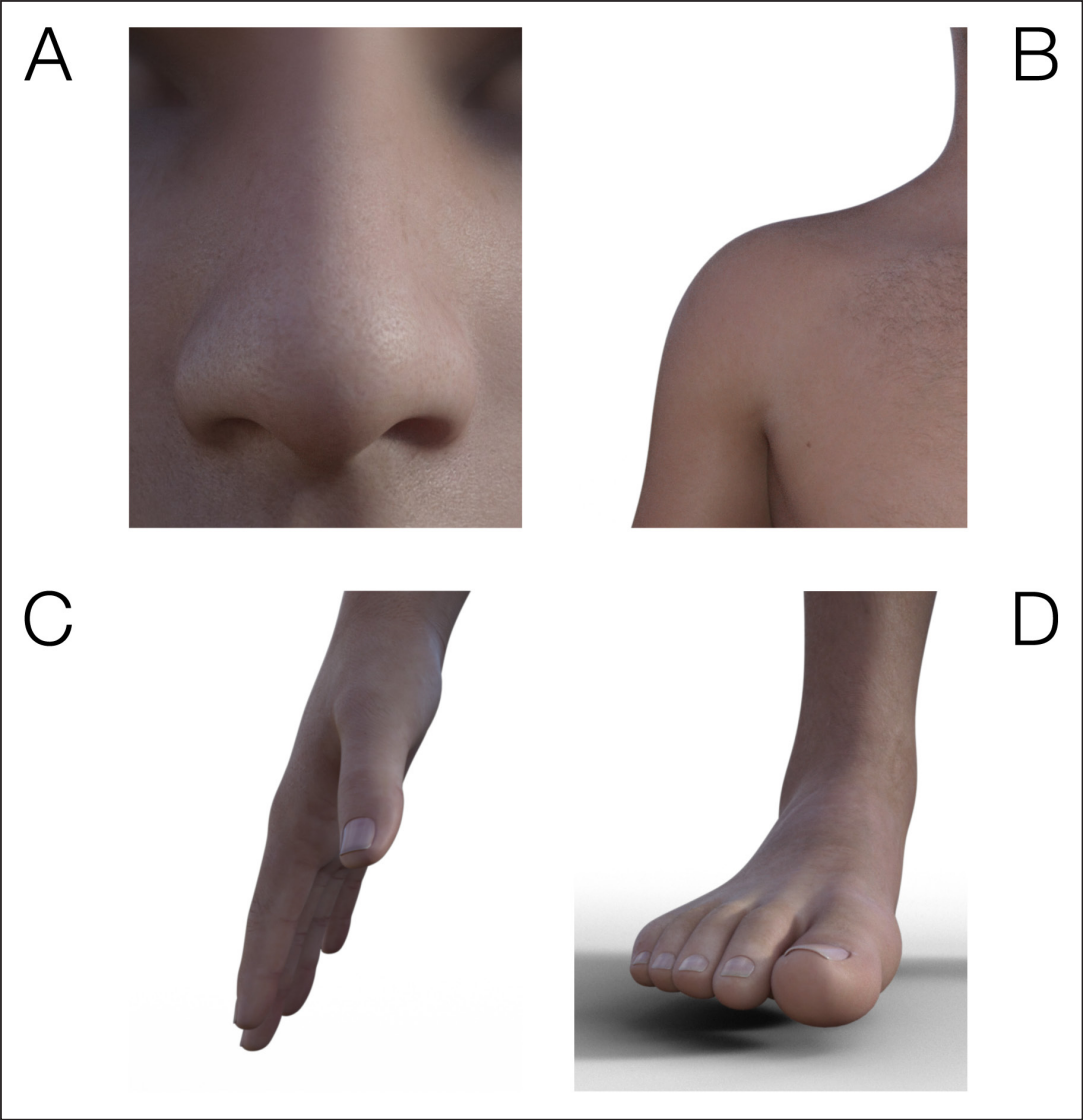
Examples of images of body parts used in Experiment 3: nose **(A)**, shoulder **(B)**, hand **(C)** and foot **(D)**. Images were obtained using DAZ^3D^ (Daz Productions, Inc; https://www.daz3d.com/).

Using DAZ^3D^ (Daz Productions, Inc; https://www.daz3d.com/) we first rendered a standard human body and then, from it, we extracted the images for the selected body parts (a few examples are reported in Figure 4 for the complete set of stimuli see: Supplementary Material).

A first pilot study (*n* = 43) was performed in order to ensure that the selected images could be consistently labeled with the relative body part originally associated. All the images reached good naming indexes (accuracy > 70%), except for the *mouth* stimulus, which 47% of the participants labeled as *lips*. We decided to keep the image in the final set of stimuli, but a control analysis was performed on the collected data in order to rule out any possible confounds (see *Data analysis* and *Results* sections).

The selected images were then paired for a total of 36 possible pairs combined across the two reference points (*eyes* or *feet*, as for Experiment 2), which were also reversed (i.e., with respect to their spatial position on the screen), for a total of 72 pairs. To increase the number of trials and hence statistical power, each possible combination appeared two times, for a total of 144 trials.

At the beginning of the experiment, participants were instructed to focus on one of the two reference points, and then, in each trial they were shown an image-pair and asked to indicate which one of the two images was closer to the reference. Participants were instructed to respond as fast and accurately as possible by pressing the left/right key (A and L) to indicate the image placed on the left side or the one on the right side. The trials were presented in random order.

The trial timeline was identical to Experiment 2 (see Figure 2). At the end of the task participants were asked to indicate in which position (i.e., standing or sitting) they imagined the body while performing the experiment.

As for Experiment 2, participants were tested online using Psychopy (Peirce, 2007, 2009; Peirce & MacAskill, 2018; Peirce et al., 2019) through the online platform Pavlovia (https://pavlovia.org/).

#### Distributional semantic model

The Italian DSM used was the same adopted in Experiment 1 and Experiment 2.

#### Computation of body and semantic predictors

The computation of body and semantic predictors was identical to Experiment 2.

#### Data analysis

Data analysis was identical to Experiment 2. The only exception was that here we further performed a control analysis including LDists predictors computed with the word *lips* instead of *mouth* in order to exclude possible confounds generated by the *mouth* image. This analysis is reported as Supplementary Material.

### Results

Trials in which overall RTs were faster than 300 ms (.007% of the trials) were excluded from the analysis. Participants did not respond to .7% of the trials. All the participants had accuracy > 80%, and the mean error rate was = 8%.

**Figure 5:**
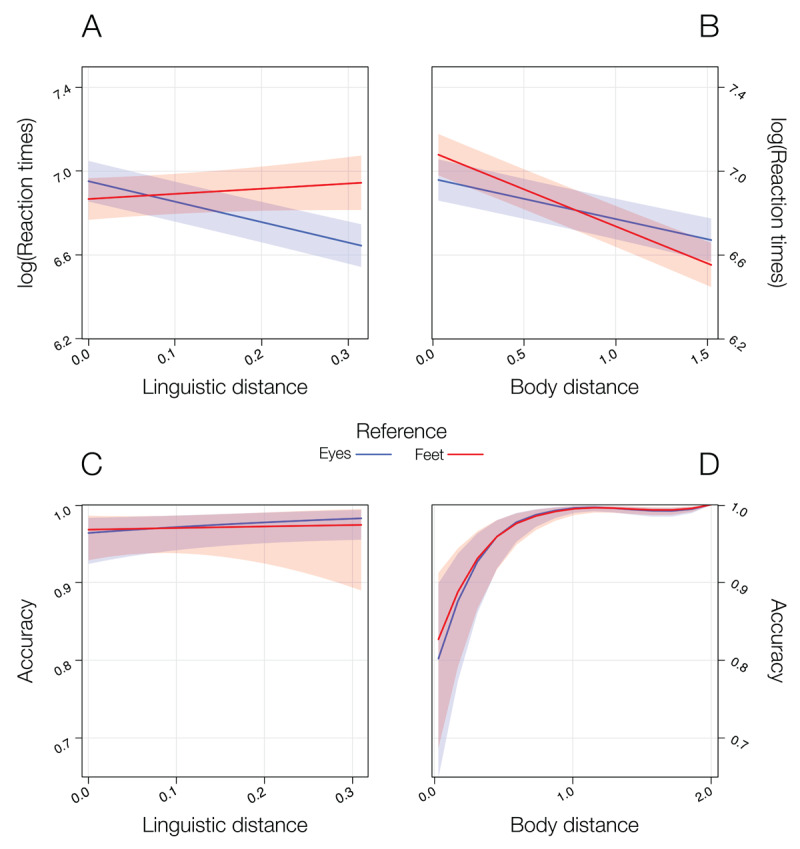
Plots of the significant effects observed in the statistical model comprising real body and linguistic distances in Experiment 3. For RTs, the higher the distances, the faster participants’ response, with this effect being moderated by the reference point. Specifically, the effect of linguistic distance is stronger in the *eyes* condition then in the *feet* condition, with the latter being not significant **(A)**, while for body distance the opposite pattern was found **(B)**. For accuracy, no effect was found for the semantic predictor **(C)**, while for the body distance predictor, the higher the distance, the higher participants’ accuracy **(D)**.

The results of the LMM on RTs and of the GLMM on accuracy are reported in Table 2, and Figure 5.

**Table 2:** Results of the LMM on RTs and of the GLMM on accuracy for Experiment 2.


FIXED EFFECT	*REACTION TIMES*			*ACCURACY*		
	
	*F*-VALUE	*NumDF, DenDF*	*p*-VALUE	*χ*^2^-VALUE	*DF*	*p*-VALUE

ΔBDist	266.12	**1,1604**	**<.001**	94.53	**1**	**<.001**

ΔLDist	13.36	**1,587**	**<.001**	2.74	**1**	.09

Reference	.01	**1,44**	.92	1.56	**1**	.21

ΔBDist : Reference	26.69	**1,1948**	**<.001**	.47	1	.49

ΔLDist : Reference	31.21	**1,478**	**<.001**	.27	1	.60


On RTs, the significant interaction ΔBDist by Reference indicated that the negative effect of ΔBDist is stronger in the *feet* condition compared with the *eye* condition, but both are significant, *b* = –.35, *t*(2344) = –14.91, *p* < .001, and *b* = –.19, *t*(1281) = –8.76, *p* < .001, respectively. Conversely, the significant interaction ΔLDist by Reference indicated that the negative effect of ΔLDist is stronger in the *eyes* condition compared with the *feet* condition, and only the former is significant, *b* = –.98, *t*(3411) = –11.06, *p* < .001, and *b* = .25, *t*(397) = 1.29, *p* = .20, respectively.

On accuracy, no interaction was found. The only significant effect was the one of ΔBDist, indicating that, the higher the distance, the higher participants’ accuracy, *b* = 4.10, *z* = 9.72, *p* < .001.

The control analysis including LDist predictors computed using the word *lips* instead of *mouth* led to the same results (see Supplementary Material).

Although here the task tapped to a greater extent onto non-linguistic visual processing, as participants were presented with images depicting body images, we found results consistent with the idea that both visual and semantic processes could be involved in body representation. Specifically, while for accuracy only the real body distance predictor was found to be significant, for response latencies, both predictors were found to be significant (body distances for both reference points and linguistic distances for eyes only). Interestingly, replicating the pattern found in Experiment 2, visual and semantic predictors were found to interact with the references point used.

## Discussion

In the present study we explored whether a high-level body map, not directly grounded on perceptual input, can be retrieved from natural language, by taking advantage of distributional semantic models (DSMs). Our findings showed that natural language embeds a body map that retains some key structural proprieties (i.e., distances between body parts) of the real body map. Perhaps more critically, in two behavioral experiments in which participants were asked to indicate which of two stimuli (either words or images) describing body parts was closer to a reference point, results showed that linguistic information concurred to explain performance along with real body distances. These findings suggest that two different sources, one mainly related to visual information, and another captured by high-level linguistic information, are activated while mentally scanning and representing the human body. More specifically, the present findings support theories arguing that mental representation relies on both perceptual and linguistic statistical information (e.g., Louwerse, 2011; 2018; 2021).

In Experiment 1, we investigated to what extent linguistic distances extracted from DSMs resemble real body distances across six different languages. Interestingly, results indicated that the model without the interaction is the best one explaining the data, and thus that the size of the effect is comparable across the languages included. This means that the structural organization of body parts is similarly represented across different languages; yet, future target studies are needed to corroborate the generalization of our results at the cross-cultural level, especially because the relatively low number of languages tested. More broadly, our findings indicate that the linguistic distance of body parts as extracted from natural language significantly resemble real body distances (i.e., with higher body distance associated with higher linguistic distance). This means that a body map can be approximated from linguistic data (e.g., Louwerse & Benesh, 2012). Complementary to this, previous studies investigating body parts dimensions have shown that these, when extracted from natural language (i.e., examining the frequencies of words referring to body parts across several languages, hence with a very different approach from the one employed here), are distorted towards the sensory homunculus rather than the actual body proportions (Günther & Rinaldi, 2022). However, notwithstanding the overall convergence between the real body map and the linguistic one, the language-based body was distorted as compared to the real body, since a good portion of variance remained unexplained.

Based on this discrepancy, in Experiment 2 we explored whether the distortions emerging from language are in line with biases in the mental representation of the human body. Specifically, participants were presented with the names of two body parts (e.g., hand – shoulder) and had to indicate the one closer to a body reference point (i.e., eyes or feet). Results showed that both linguistic and real body distances predicted participants’ response latencies and accuracy. Specifically, for both predictors, the lower the distance, the higher participants’ response latencies, with this effect being stronger for the eyes as a reference point for the linguistic predictor, and for the feet as a reference point for the real distance predictor. The higher real body distance effect when the feet were taken as a reference point may be interpreted by considering the standard way we explore and scan human bodies. We indeed preferentially start scanning the body from the eyes, as this is the most prominent reference point for various reasons (e.g., we see through the eyes, they are important for nonverbal communication, etc.). As such, the higher distance effect when the feet were taken as the reference point, may reflect an enhanced visual scanning strategy because this is the less canonical direction for exploring the human body. On the contrary, the body seems to be better represented in language by taking the eyes as the reference point, as indexed by the strongest linguistic distance effect in this condition. Finally, for accuracy, the lower the linguistic and real distance, the lower participant’s accuracy (with this effect not being modulated by the reference point). Together, Experiment’s 2 results, indicate that both linguistic and visual processes are mutually involved in body representation.

Nevertheless, one could argue that the stimuli used in Experiment 2 (i.e., body part names) could have over-emphasized the observed linguistic involvement. To control for this possibility, in Experiment 3 a new sample of participants was tested using visual stimuli. Participants were presented with the images of two body parts and had to indicate which one was closer to a reference point (i.e., eyes or feet). Replicating results from Experiment 2, in Experiment 3 we found that, for response latencies, the lower the distance the higher participants’ response latencies, with this effect being significant for the eyes reference point for the linguistic predictor but not for the feet reference point, while for the real body distance predictor results were fully consistent with Experiment 2. These results largely replicate Experiment 2 and support our interpretation on the dissociation found there. Specifically, we can speculate that the stimuli used in Experiment 3 (i.e., images) require a more prominent visual processing, thus we could have expected vision-based effects to be even stronger as compared with Experiment 2. Our results support this view, with participants not relying on language information in the “feet” condition, as visual information is likely enough to solve the task. Consistent with Experiment 2, then, the real body distance effect found during the “eyes” condition is smaller as compared with the “feet” condition. For accuracy, only the real distance index significantly predicted participants’ behavior, with an effect comparable with the one found in Experiment 2.

In interpreting our findings, several clarifications on the methodology adopted in Experiment 2 and Experiment 3 should be made. Firstly, it should be noted that we used body distances as extracted from a 2D (instead of 3D) representation, since the body parts selected are on the surface. It is therefore likely that the sagittal plane would bring only limited information on bodily distances at the ones considered in this study. Secondly, as the distances between body parts (can) change dramatically depending on the posture of the body. For this very reason, participants were asked to indicate in which position they imagined the body while performing the task and the metrics extracted from that position were used to predict human performance. Yet, future studies are needed to probe whether the reliance on perceptual vs. linguistic information in tasks tapping on bodily parts distances may differ depending on the specific posture imagined by participants.

Previous studies interpreted the presence of an analogous distance effect when processing body parts stimuli, by tracing it back to mental imagery strategies (Van Elk & Blanke, 2011). That is, participants may scan a mental image of the body while performing the task, hence in turn relying on visuo-spatial experience with the body (e.g., Noordzij & Postma, 2005; Peviani et al., 2019; Struiksma et al., 2011). Following this rationale, a visual imagery strategy may have been adopted in Experiment 2 and, on an even higher extent, in Experiment 3. Consistent with this possibility, we found that the real distance between body parts explained human behavior across both experiments. Yet, and crucially, our findings demonstrate that when humans explore their body, they do not rely only on a visual imagery strategy. Rather, higher-level symbolic information (as extracted from natural language) about the human body do systematically contribute to judge the distance between body parts. Notably, this high-level body map contributes to human body representation over and above the visual body map, indicating that the former cannot be simply conceived as a by-product of perceptual experience. In support to this view, this pattern was replicated across two behavioral experiments, with the second one directly tapping on visual processing (i.e., participants were shown with body images). Consistent with our results, previous studies reported that conceptual properties and linguistic information as extracted from natural language do play a role in humans’ body representation (Bracci et al., 2015; Tillman et al., 2012). Critically, in the present study we did not just employ word frequencies (or the frequency of word co-cooccurrences), but rather an index derived from DSMs data that allows to estimate words meaning in terms of its usage in natural language. Additionally, this study thus testifies the simultaneous activation of two distinct body maps, one relying on perceptual and one on linguistic experience.

The present findings contribute to the current debate on mental representations and, more generally, on the role of semantic memory in complex human behavior. Regarding the former, our findings indicate the existence of two (partially) independent (body) representation processes. Regarding the latter, previous studies have reported similar results (in terms of linguistic experience) in the geographical representation domain (Gatti, Marelli et al., 2022; Louwerse & Zwaan, 2009), and the present study extends such evidence to the body representation domain, indicating that humans are likely relying on linguistic information too while performing a broad range of spatial tasks. In other words, our findings support the view that we get to know “the meaning of a word [or the location of a body part] by the linguistic and perceptual company the word keeps” (Louwerse, 2018, p. 573).

In conclusion, across three experiments, we provide evidence of the existence of multiple experiential traces concurring to body representation. The first trace is reliant on sensorimotor experience and has been largely recognized in prior literature as one of the major sources for constructing a human body representation, in line with embodied perspectives (e.g., Barsalou, 2008). The second trace, reliant on symbolic and higher-level experiences, had remained until now elusive within the body representation domain. Here, we demonstrate that – when properly quantified – the participation of this higher-level map is crucial for a better grasping of human body representation. By revealing the complex factors that dynamically contribute to the representation of the human body, this study clarifies more generally the deep entanglement between perceptual and symbolic learning.

## Data Accessibility Statement

All data, scripts and codes used in the analysis are available at: https://osf.io/dnt2p/. This study was not preregistered.

## Footnotes

We measured in Adobe Photoshop CS (2004) the height of the human figure and then we computed a proportional index, starting from the distance of each pair in the figure (measured in pixels).

The choice to use ΔAIC = 2 as boundary was made following the approach proposed by Wagenmakers and Farrell (2004), based on Akaike weights. A ΔAIC = 2 would indicate that the model with lower AIC is 2.7 times more likely to be a better model (in terms of Kullback-Leibler distance from the “real” distribution) than the model with higher AIC.

We decided to use the two words in their plural form in order to compute the BDist measures from the average point between the two eyes or between the two feet across the two conditions.

In order to investigate if the body position imagined (a sitting body was imagined in 13 of 80 sessions) modulated the effect of the BDist predictors, we estimated a LMM having the ΔBDist predictor and its interaction with the body position imagined as fixed factors. The interaction effect was not significant, *p* = .94, thus the body position imagined was dropped from the following estimated models. Similarly, no interaction effect was found in the GLMM on accuracy, *p* = .35.

In this case we opted for removing those who imagined the body as sitting due to their low numerosity which would have prevented us to investigate with good reliability possible interaction effects due to the body position imagined.

## Prior dissemination

A previous version of this article was uploaded on PsyArXiv (https://psyarxiv.com/mt3rh).
